# Neighborhood Deprivation and the Effectiveness of Mobile Health Coaching to Improve Periconceptional Nutrition and Lifestyle in Women: Survey in a Large Urban Municipality in the Netherlands

**DOI:** 10.2196/11664

**Published:** 2019-04-11

**Authors:** Dionne V Gootjes, Matthijs R van Dijk, Maria PH Koster, Sten P Willemsen, Eric AP Steegers, Régine PM Steegers-Theunissen

**Affiliations:** 1 Department of Obstetrics and Gynecology Erasmus Medical Center University Medical Center Rotterdam Rotterdam Netherlands; 2 Department of Biostatistics Erasmus Medical Center University Medical Center Rotterdam Rotterdam Netherlands

**Keywords:** pregnancy, telemedicine, lifestyle, nutritional status, preconception care

## Abstract

**Background:**

In 2011, we launched the Smarter Pregnancy mobile health (mHealth) coaching program, which has shown to effectively improve inadequate nutrition and lifestyle behaviors in women before and during pregnancy. It is known that in deprived neighborhoods, risk factors for adverse pregnancy outcomes like inadequate nutrition and lifestyle behaviors accumulate. However, it has not yet been investigated whether the Smarter Pregnancy program is equally effective in women living in deprived neighborhoods.

**Objective:**

This paper aimed to study the associations between neighborhood deprivation and improvement of inadequate nutrition and lifestyle behaviors of women who were either contemplating pregnancy or already pregnant and subscribed to the Smarter Pregnancy program.

**Methods:**

We performed an additional analysis on data from women who used the Smarter Pregnancy program from 2011 to 2016. The program comprised 24 weeks of coaching on 5 nutrition and lifestyle behaviors, of which adequate intakes or lifestyle behaviors were defined as an intake of 200 grams or above of vegetables, 2 pieces of fruit, daily folic acid supplement use of 400 µg per day, and no smoking or alcohol consumption. Neighborhood deprivation was determined according to the status scores of the Netherlands Institute for Social Research. Logistic regression analyses and generalized estimating equation models were used to assess the associations between the neighborhood status score (NSS) and the improvement of inadequate nutrition and lifestyle behaviors, taking into account the behaviors at baseline. We adjusted the analyses for maternal age, body mass index, geographic origin, pregnancy status, and participation as a couple.

**Results:**

Of the 2554 women included, 521 participated with their male partner. Overall, daily vegetable intake was most frequently inadequate at the start of the program (77.72, 1985/2554). Women with a higher NSS (ie, nondeprived neighborhood) smoked less often (adjusted odds ratio [OR] 0.85; 95% CI 0.77-0.93), consumed alcohol more often (adjusted OR 1.14, 95% CI 1.04-1.24), and were less likely to complete the 24 weeks of coaching (OR 0.91, 95% CI 0.88-0.95) compared with women who lived in a neighborhood with a low NSS (ie, deprived). In the total group, the relative improvement of inadequate nutrition and lifestyle behaviors after 24 weeks of coaching was between 26% and 64%. NSS was negatively associated with this improvement, indicating that women with a higher NSS were less likely to improve inadequate nutrition and lifestyle behaviors, especially vegetable intake (adjusted OR 0.89, 95% CI 0.82-0.97).

**Conclusions:**

The Smarter Pregnancy mHealth coaching program empowers women to improve inadequate nutrition and lifestyle behaviors. Unexpectedly, the program seemed more effective in women living in deprived neighborhoods. It is important to unravel differences in needs and behaviors of specific target groups to further tailor the mHealth program on the basis of demographic characteristics like neighborhood deprivation.

## Introduction

### Background

Worldwide, there are substantial differences in perinatal morbidity and mortality rates between and within countries, which may indicate inequalities in perinatal as well as population health [1,2]. Several underlying factors can explain these differences such as maternal-specific (eg, age, body mass index, BMI, and parity), environmental (eg, air pollution and extreme temperature), and community-derived (eg, housing conditions and poverty) factors [3-6]. As in other countries, perinatal morbidity and mortality rates in the Netherlands also differ among districts, with particularly high mortality rates in the country’s 4 largest cities. This is mainly because of the large number of deprived neighborhoods in these cities [7-9].

Risk factors for adverse pregnancy outcomes, such as poor nutrition, lifestyle and housing conditions as well as lower health literacy, often accumulate in residents of deprived neighborhoods [6,9,10]. However, living in a deprived neighborhood itself has also been described as an independent risk factor for poor health outcomes [11]. Exposure to the abovementioned risk factors during the periconception period (ie, the 14 weeks before conception until 10 weeks after conception) [12,13] can have a detrimental effect on maternal and neonatal outcome. Moreover, on the longer term, the effect of these adverse outcomes is not limited to perinatal health; it also extends to the child’s health later in life [14,15]. Therefore, it is important to change inadequate nutrition and lifestyle behaviors during the periconception period.

According to the transtheoretical model of behavioral change, intentional behavioral change can be achieved after passing 6 different stages, from precontemplation to maintenance and termination [16].

However, behavioral change is more challenging for individuals who have limited health literacy or impaired financial resources, who are less educated, and live in more deprived neighborhoods [3,17,18]. From this background, we hypothesize that women who live in more deprived neighborhoods are less likely to improve inadequate nutrition and lifestyle behaviors before and during pregnancy compared with women who live in less deprived neighborhoods.

Currently, mobile health (mHealth) apps are widely available and used for health improvement. mHealth apps can be designed to a specific population and target of interest and may be offered anytime and anywhere at low costs. Therefore, mHealth is a promising medium to support people to improve nutrition and lifestyle behaviors [19,20]. In 2011, after more than 30 years of research on the impact of nutrition and lifestyle behaviors on reproduction, we developed and launched the Smarter Pregnancy mHealth coaching program [21] for women, together with their male partners, who are contemplating pregnancy or are already pregnant [22,23]. Smarter Pregnancy is a Web-based program that can be used on a mobile device, comprising screening questions, thereafter comprising personal coaching through short message service (SMS) and email (Multimedia Appendix 1).

Previously, van Dijk et al analyzed survey data of all subscribers to the Smarter Pregnancy program to assess compliance, feasibility, usability, and first effectiveness of the program. It was shown that the program contributes to significant improvements of inadequate nutrition and lifestyle behaviors—that is, vegetable (+26%) and fruit intake (+38%), folic acid supplement use (+56%), smoking (−35%), and alcohol consumption (−42%)—in couples before and during pregnancy, which also resulted in an enhanced pregnancy chance in both fertile and subfertile couples up to 40%. Besides, a high compliance (65%) and usability were reported [22,24].

### Objectives

However, it has not yet been investigated whether the Smarter Pregnancy program is equally effective in women who live in deprived neighborhoods. Therefore, our current aim was to investigate in an additional analysis, associations between neighborhood deprivation and the improvement of inadequate nutrition and lifestyle behaviors of women before and during pregnancy who previously subscribed to the Smarter Pregnancy program.

## Methods

### Study Design

We used the data of an epidemiological survey conducted among all women who subscribed to the Smarter Pregnancy program for an additional analysis [21]. Women and their partners living in the Netherlands were invited to subscribe to the Smarter Pregnancy program. Inclusion criteria were the following: aged between 18 and 45 years, an active wish to contemplate pregnancy, pregnant less than 13 weeks, the possession of a mobile phone with internet access, and a sufficient knowledge or understanding of the Dutch language. For male partners, the same inclusion criteria had to be met but without an upper age limit. Registration to this mHealth program was recommended to patients who visited the Division of Reproductive Medicine of the Department of Obstetrics and Gynecology of the Erasmus Medical Centre (MC) and to women who attended a community midwife in the Rotterdam area. However, as the website had an open access policy, other visitors were able to register. Although women could either participate alone or together with their male partner, because of the small sample size of couples, in this study, we only analyzed data from women, and participation as a couple was taken into account as a covariate.

The coaching model developed for the Smarter Pregnancy program is based on the most recent knowledge on the effect of vegetable, fruit, and folic acid supplement intake, smoking and alcohol consumption on pregnancy chance, course, and outcome [18,25,26]. For the content of the platform, the stage of the model of Prochaska and Diclemente’s was taken into account, which describes the readiness for behavioral change. This was implemented by informing participants about the positive effects of adequate nutrition and lifestyle behaviors on pregnancy course and outcome, which could affect the readiness to improve these behaviors [16]. Characteristics of the attitude, social influence, and self-efficacy model were implemented by enabling individuals as well as their partners to improve behavior [27]. Fogg's behavior model was applied by including triggers throughout the program to support motivation and thereby increase the ability to change nutrition and lifestyle behaviors [28]. Furthermore, the Smarter Pregnancy program meets the highest rules of legislation for medical devices in Europe, and it received the Conformité Européenne, classe 1 classification (2013). Effectiveness of the program has previously been demonstrated and described by van Dijk et al [24].

### Intervention

The coaching program starts with a baseline screening on nutrition (ie, vegetable and fruit intake and folic acid supplement use) and lifestyle (ie, smoking and alcohol consumption) behaviors that significantly affect fertility and pregnancy course and outcome [24]. The mHealth coaching lasts for a period of 24 weeks and only targets the nutrition and lifestyle behaviors that are inadequate at the start of the program. Coaching comprises a maximum of 3 interventions per week, comprising SMS text messages and email messages containing recommendations, vouchers, and seasonal recipes. Follow-up screening takes place at 6, 12, 18, and 24 weeks after registration (Multimedia Appendix 2). Besides nutrition and lifestyle behaviors, there are additional questions addressing pregnancy status and BMI. The technical programming is executed by Peercode BV. A detailed description of the content of the Smarter Pregnancy program has previously been published by van Dijk et al [22].

### Data Collection

Data were collected through the Smarter Pregnancy program itself. Demographic characteristics and anthropometric measurements of the participants were retrieved from the Smarter Pregnancy database—zip code, sex (male or female), age (continuous), pregnancy status (pregnant or not pregnant), and BMI (calculated from self-reported height and weight). Geographic origin was not reported by participants themselves. Therefore, we used the surnames of the participants to ascribe them a geographic origin, a method that is considered valid when self-identification is not available [29]. Classification was performed by 3 investigators (DG, MRD, and MPHK) who separately categorized all participants’ surnames into 2 groups, that is, Western (Europe, excluding Turkey, North America, Oceania, Indonesia, and Japan) and non-Western (Africa, Latin America, Asia excluding Indonesia and Japan, and Turkey) origin. Any disagreement was resolved by discussion among the 3 investigators, which was the case within 7.6% (195/2554) of the surnames.

### Outcomes

Compliance to the Smarter Pregnancy program was defined as the percentage of participants who filled in the last questionnaire of the program after 24 weeks of coaching. At baseline and after 24 weeks of coaching, the reported nutrition and lifestyle behaviors were classified as adequate or inadequate. Adequate behavior was defined as a daily intake of at least 200 grams of vegetables and at least 2 pieces of fruit, daily folic acid supplement use of at least 400 µg starting before conception and lasting until the 12th week of pregnancy, and no smoking or alcohol consumption [30].

To adjust for nutrition and lifestyle behaviors, a total risk score (TRS) was calculated. For vegetable and fruit intake, folic acid supplement, and alcohol use, 0 points were assigned in case a participant had an adequate intake or use [24]. For inadequate intake or use, 3 points were assigned. For smoking, 6 points were assigned in case of inadequate use, because of its known strong negative impact on pregnancy course and outcome [25,31]. Consequently, TRS in this study ranges from 0 (most adequate) to 18 (most inadequate).

To assign participants a neighborhood deprivation state, the status scores of the Netherlands Institute for Social Research were used. These scores follow a standard normal distribution by design and are calculated for all 4-digit zip codes in the Netherlands on the basis of 4 neighborhood characteristics—the average income, the number of nonemployed residents, the number of lower educated residents, and the number of households with a low income [32]. When the neighborhood status score (NSS) is low, this indicates a deprived neighborhood. A high NSS indicates a nondeprived neighborhood [33]. Since 1998, NSS is calculated every 4 years. For this study, the NSS of the year 2014 was used to determine the classification of the neighborhood participants lived in while using the Smarter Pregnancy program. In 2014, the interquartile range (IQR) of the NSS in the Netherlands was –0.57 to 0.71.

### Data Analysis

All participants who started the program were included in the analysis at baseline. However, improvement of nutrition and lifestyle behaviors was only examined in those individuals who scored inadequate at any of these behaviors at the start of the program. To minimize selection bias, multiple imputation using chained equations was performed to handle missing data of women who prematurely resigned from the program. For those women, it was assumed that the adequacy of their nutrition and lifestyle behaviors at the last reported screening moment would not have changed until the end of the program (24 weeks).

Univariate linear and logistic regression analysis was used to study associations between demographic characteristics of the study population (maternal age, BMI, geographic origin, pregnancy status, whether a woman participated as a couple or alone, and TRS) and the NSS at the start of the program. Logistic regression analysis was used to examine the association between the NSS and (in) adequate nutrition and lifestyle behaviors at the start of the program. To study the improvement of inadequate nutrition and lifestyle behaviors after 24 weeks of coaching, generalized estimating equations with an independent working correlation matrix were used to model the fraction of the study population that scored inadequate at baseline, taking into account that less improvement may be expected when less women show inadequate behavior at baseline. Interaction tests were performed to study interactions of geographic origin, participation as a couple, or being pregnant at the start of the program on the association between NSS and nutrition and lifestyle behaviors.

Statistical analyses were performed using SPSS version 21 software package (IBM Corp) and R version 3.4 (Foundation for Statistical Computing). *P*<.05 values were considered statistically significant. No alpha adjustment for multiple comparisons was made.

### Ethical Approval

Details of ethical approval included the following: This survey was conducted according to the guidelines laid down in the Declaration of Helsinki. All procedures involving patients were approved by the Medical Ethical and Institutional Review Board of the Erasmus MC, University Medical Centre, Rotterdam, the Netherlands (MEC-2011–524, approved on 22 December 2011). Digital informed consent was obtained from all participants.

## Results

### General Characteristics

**Figure 1:**
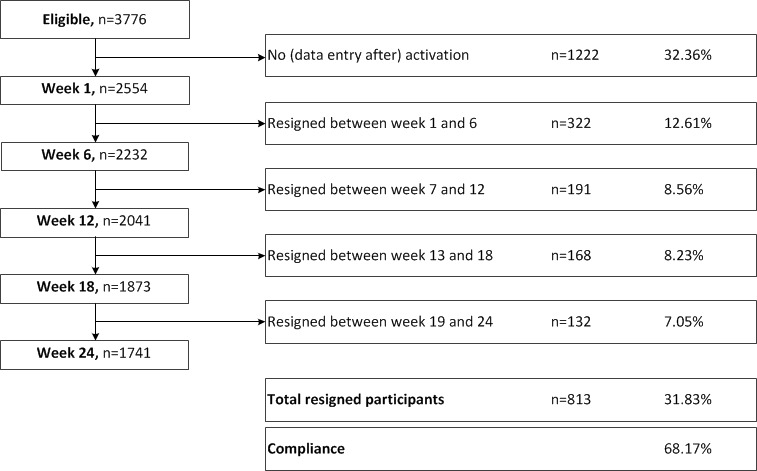
Flowchart of study participants that completed or resigned from the Smarter Pregnancy mobile health coaching program.

A total of 3776 women registered to the Smarter Pregnancy program, out of which 32.36% (1222/3776) of the women were excluded because of absence of activating the registration, incomplete registration, or incomplete data entry at the start of the program (Figure 1). Consequently, a total of 2554 women were included in the analysis, out of which 521 participated with their male partner. The median age of women at the start of the program was 31 years and most women were of Western geographic origin (72.91% (1862/2554)). Of all nutrition and lifestyle behaviors, daily vegetable intake was most frequently inadequate at the start of the program (77.72%(1985/2554) Table 1).

Women with a higher NSS (ie, who lived in a less deprived neighborhood) were older (beta=.04; 95% CI 0.03-0.05) and more often participated as a couple (beta=.18; 95% CI 0.11-0.25). Moreover, these women were more often pregnant at the start of the program (beta=−.30; 95% CI −0.41 to −0.19), had a lower BMI (beta=−.03; 95% CI −0.04 to −0.02), and were less often of non-Western geographic origin (beta=−0.78; 95% CI −0.85 to −0.70; Table 2).

Compliance to the Smarter Pregnancy program was 68.17% (1741/2554; Figure 1). Women with a higher NSS were less likely to finish the 24 weeks of coaching (odds ratio [OR] 0.91, 95% CI 0.88-0.95).

**Table 1:** Demographics of the study population and nutrition and lifestyle behaviors at the start and after 24-weeks of coaching with the Smarter Pregnancy mobile health coaching program (N=2554).

Characteristics			Statistics
**Demographics**			
	Age^a^ (years), median (IQR^b^)		31 (28 to 34)
	Neighborhood status score, median (IQR)		−0.18 (−1.14 to 0.69)
	Pregnant at baseline (yes), n (%)		1300 (50.90)
	Body mass index^a^ (kg/m^2^), median (IQR)		23.9 (21.4 to 27.5)
	Participating as couple (yes), n (%)		521 (20.4)
	Geographic origin^a^ (Western), n (%)		1862 (72.91)
**Nutrition and lifestyle behaviors**			
	**Vegetable intake (inadequate), n (%)**		
		At start of the program	1985 (77.72)
		At 24 weeks	1462 (57.24)
	**Fruit intake (inadequate), n (%)**		
		At start of the program	1024 (40.09)
		At 24 weeks	576 (22.6)
	**Folic acid supplement use (inadequate), n (%)**		
		At start of the program	316 (12.4)
		At 24 weeks	114 (4.5)
	**Smoking (yes), n (%)**		
		At start of the program	252 (9.9)
		At 24 weeks	182 (7.1)
	**Alcohol consumption (yes), n (%)**		
		At start of the program	605 (23.7)
		At 24 weeks	339 (13.3)
**Total risk score, median (IQR)**			3 (1-6)

^a^Age, body mass index, and geographic origin were missing in 1.2%, 0.4%, and 9.6% of the study population, respectively.

^b^IQR: interquartile range.

**Table 2:** Univariate associations between the neighborhood status score and demographic factors (N=2554).

Characteristic	Β^a^ (95% CI)	*P* value
Age^b^ (years)	0.04 (0.04 to 0.05)	<.001
Pregnant at baseline (yes)	−0.30 (−0.41 to −0.19)	<.001
Body mass index^b^ (kg/m^2^)	−0.03 (−0.04 to −0.02)	<.001
Participating as couple (yes)	0.18 (0.11 to 0.25)	<.001
Geographic origin^b^ (non-Western)	−0.78 (−0.85 to −0.70)	<.001
Total risk score	−0.01 (−0.02 to 0.001)	.42

^a^β: effect size.

^b^Age, body mass index, and geographic origin were missing in 1.2%, 0.4%, and 9.6% of the study population, respectively.

**Table 3:** The association between the neighborhood status score and inadequate nutrition and lifestyle behaviors in all participating women at the start of the program (N=2554).

Nutrition and lifestyle behaviors	Crude, OR^a^ (95% CI)	*P* value	Adjusted^b^, OR (95% CI)	*P* value
Vegetable intake (inadequate)	1.04 (0.98-1.11)	.21	1.04 (0.98-1.12)	.20
Fruit intake (inadequate)	1.03 (0.97-1.09)	.29	1.01 (0.95-1.07)	.74
Folic acid supplement use (inadequate)	1.00 (0.92-1.08)	.94	1.00 (0.90-1.09)	.85
Smoking (yes)	0.85 (0.78-0.92)	<.001	0.85 (0.77-0.93)	<.001
Alcohol consumption (yes)	1.23 (1.15-1.32)	<.001	1.14 (1.04-1.24)	.004

^a^OR: odds ratio.

^b^Adjusted for body mass index, age, geographic origin, pregnancy status, and participation as a couple.

**Table 4:** The association between the neighborhood status score and improvement of inadequate nutrition and lifestyle behaviors after 24 weeks of coaching in all women who scored inadequately at the start of the mobile health program.

Nutrition and lifestyle behaviors	Crude, OR^a^ (95% CI)	*P* value	Adjusted^b^, OR (95% CI)	*P* value
Vegetable intake (inadequate) (n=1462)	0.86 (0.79-0.94)	.001	0.89 (0.82-0.97)	.02
Fruit intake (inadequate) (n=576)	0.90 (0.81-1.00)	.051	0.93 (0.84-1.04)	.21
Folic acid supplement use (inadequate) (n=114)	1.00 (0.80-1.24)	.97	1.02 (0.80-1.30)	.87
Smoking (yes) (n=182)	0.87 (0.69-1.10)	.23	0.90 (0.69-1.16)	.40
Alcohol consumption (yes) (n=339)	1.04 (0.9-1.19)	.57	1.05 (0.91-1.21)	.49

^a^OR: odds ratio.

^b^Adjusted for body mass index, age, geographic origin, pregnancy status and participation as a couple.

### Nutrition and Lifestyle Behaviors

As coaching was only aimed at nutrition and lifestyle behaviors that were reported as inadequate at the start of the program, improvement of these behaviors was only studied in subsets of women. Overall, women who used the Smarter Pregnancy program improved all nutrition and lifestyle behaviors (Table 1). At the start of the program, vegetable intake was most frequently inadequate (77.72% (1985/2554)). After 24 weeks of coaching, this was reduced to 57.24% (1462/2554), which is a relative improvement of 26%. The largest improvement (relative improvement of 64%) was achieved for folic acid supplement use; this was inadequate in 12.4% (316/2554) women at the start of the program and reduced to 4.5% (114/2554) after 24 weeks.

At the start of the program, no statistically significant association between NSS and inadequate vegetable and or fruit intake was found. However, women with a higher NSS were significantly less likely to smoke (adjusted OR 0.85; 95% CI 0.77-0.93) but more likely to consume alcohol (adjusted OR 1.14, 95% CI 1.04-1.24; Table 3). NSS was not associated with the amount of improvement in smoking and alcohol consumption after 24 weeks of coaching (Table 4). However, NSS was significantly negatively associated with improvement of vegetable intake after 24 weeks of coaching—women with a higher NSS improved their vegetable intake less than women with a lower NSS (adjusted OR 0.89, 95% CI 0.82-0.97). Improvement of the other nutrition and lifestyle behaviors did not significantly depend on NSS (Table 4).

Interaction tests showed that the association between NSS and nutrition and lifestyle behaviors was not significantly different in women who did or did not participate as a couple and who were pregnant or not pregnant at the start of the program. However, at the start of the program, the association between NSS and alcohol consumption was stronger in non-Western (adjusted OR 1.74, 95% CI 1.33-2.28) compared with Western women (adjusted OR 1.12; 95% CI 1.00-1.25). This difference between non-Western and Western women was not observed for the association between the NSS and improvement of alcohol consumption.

## Discussion

### Principal Findings

Following the results of van Dijk et al, this study demonstrated that women improve their inadequate nutrition and lifestyle behaviors after 24 weeks of mHealth coaching using the Smarter Pregnancy program [22]. However, especially with regard to vegetable intake, this improvement is less in women living in a lesser deprived neighborhood (higher NSS). Although women with a higher NSS were less likely to smoke and more likely to consume alcohol at the start of the program compared with women with a lower NSS, we observed no significant differences in the amount of improvement of these lifestyle behaviors. Furthermore, NSS was significant and negatively associated with compliance to the Smarter Pregnancy program; women with a higher NSS were less likely to complete the 24 weeks of coaching than women with a lower NSS.

### Comparison With Previous Work

Currently, a growing number of mHealth apps are developed for personal lifestyle and medical health care support. These apps provide interaction and targeted information on particular domains for specific target groups, and improvement in self-reported health behaviors because these apps are observed. Specifically, decreased tobacco use, increased vitamin intake, and more frequent healthy food intake have been reported after coaching by apps designed to encourage healthy behavior. Therefore, in our opinion, it is important to conduct profound research both in low- and middle- as well as in high income countries before these apps can be implemented in medical health care [34].

In this study’s population, women who lived in less deprived neighborhoods were less likely to smoke but more likely to consume alcohol, which is in line with previous studies [9,35,36]. Despite recent studies stating that residents who live in deprived neighborhoods are difficult to motivate to change unhealthy behaviors [3,17,18], in this study, those women were more likely to complete the 24 weeks of mHealth coaching and improve their nutrition and lifestyle behaviors more than women who live in less deprived neighborhoods. This is rather surprising as we expected the opposite, namely that higher educated women, more often living in a neighborhood with a higher NSS, have generally higher health literacy skills compared with women from a neighborhood with a lower NSS and therefore improve behaviors more quickly [37]. An explanation may be that higher educated women believe that they already have healthy behaviors and do not need to change [37,38]. In addition, our previously conducted focus group study among women participating in the Smarter Pregnancy program reported that higher educated women showed a lower compliance and appreciated the program less than middle- and low-educated women, who often live in neighborhoods with lower NSS [39]. This is in line with the fact that the content of the coaching is compiled so that it matches the skills and knowledge of the largest population of middle- and low-educated women who generally have a higher prevalence of unhealthy lifestyle behaviors.

### Strengths and Limitations

Strengths of this study are the large number of included participants (N=2554), the high overall compliance of 68.17% (1741/2554) of women who completed the 24 weeks of coaching, the fact that several potential confounders were taken into account in the adjusted models, and the imputation of missing data. In this study, NSS—based on a well-defined index—was used as a proxy for socioeconomic health inequality among neighborhoods. This continuous measure of neighborhood deprivation was used instead of a dichotomous measure (ie, deprived vs nondeprived), which provides a more precise evaluation of the effect of neighborhood deprivation. The use of area-based indices as a proxy for socioeconomic health is well supported in the literature; thus, the used neighborhood deprivation index can be considered a valid indicator [40,41]. NSS is a measure based on factors that are specific for (the residents in) that particular neighborhood. Indeed, we found that NSS is a representative measure for deprivation characteristics on the individual level; in less deprived neighborhoods, participating women had a lower BMI and were more likely to be of non-Western geographic origin. Furthermore, the distribution of NSS in this study cohort (IQR −1.14 to 0.69, data not shown) was comparable with the national NSS in the year 2014 (IQR −0.57 to 0.71).

Despite the fact that the inclusion period of the study population and the coaching with the Smarter Pregnancy program covers several years, the NSS of 2014 was used as the measure of neighborhood deprivation for the whole study population. As the NSS and the ratio of score among the neighborhoods do not change much over time, we consider this a valid determinant of the neighborhood deprivation within the study population.

Although geographic origin is known to be a potential confounding variable for associations with deprivation, information regarding geographic origin was not directly available from our database. To take geographic origin into account, we retrospectively performed geographic classification. This approach is considered a valid method for ascribing individuals to geographic groups when self-identification is not available [29], but unfortunately, it does not permit any further subdivision into more specific geographic groups besides Western and non-Western.

Limitations of this study are the absence of validation of nutritional status by biomarkers and the absence of a control group, although this is inherent to this study’s design. Furthermore, the Smarter Pregnancy program was only available in Dutch and on multiple devices with internet access and preferably a mobile phone. Consequently, only those familiar with the Dutch language and in possession of a mobile phone with internet access participated. Over 95% of all women and men of reproductive age living in the Netherlands have internet access on their mobile phone, making the program properly accessible [42]. However, a selection may have occurred of only those familiar with the Dutch language, who are mainly of Western origin. This is reflected by the fact that over 80% of the women in this study were of Western geographic origin, although, on the basis of the population distribution of the city of Rotterdam, a percentage of 62% was to be expected [43]. Misclassification of the geographic origin because of incorrect assignment cannot be excluded. However, the surname-based method for ascribing individuals to geographic groups when self-identification is not available is previously described as a valid method. Another form of selection bias may have been induced as the Smarter Pregnancy program was not routinely used or recommended as part of (pre) pregnancy care, and participants mostly subscribed upon their own initiative. Therefore, women could have been mainly women who are already intrinsically motivated to change nutrition and lifestyle behaviors before starting the mHealth program. Together, these limitations may contribute to the generalizability of this study’s results.

### Conclusions and Future Perspectives

Overall, we can conclude that the Smarter Pregnancy mHealth coaching program is able to motivate and support women from more and less deprived neighborhoods to improve their nutrition and lifestyle behaviors. However, women who live in more deprived neighborhoods seem to improve their nutrition and lifestyle behaviors more compared with women from less deprived neighborhoods.

Together, these findings underline the need for a more tailored version of the program, adapted to the needs of its participants on the basis of demographic characteristics, so that the program can adequately and optimally empower all women to improve their nutrition and lifestyle behaviors.

## Appendix

Multimedia Appendix 1 Smarter Pregnancy program.

Multimedia Appendix 2 Description of the coaching program.

## Abbreviations

BMI: body mass index
IQR: interquartile range
MC: Medical Centre
mHealth: mobile health
NSS: neighborhood status score
OR: odds ratio
SMS: short message service
TRS: total risk score
