# Supplementary material for: Neighborhood Deprivation and the Effectiveness of Mobile Health Coaching to Improve Periconceptional Nutrition and Lifestyle in Women: Survey in a Large Urban Municipality in the Netherlands
Source: JMIR Mhealth Uhealth. 2019 Apr 11;7(4):e11664. doi: 10.2196/11664 (PMC6482404; doi:10.2196/11664)

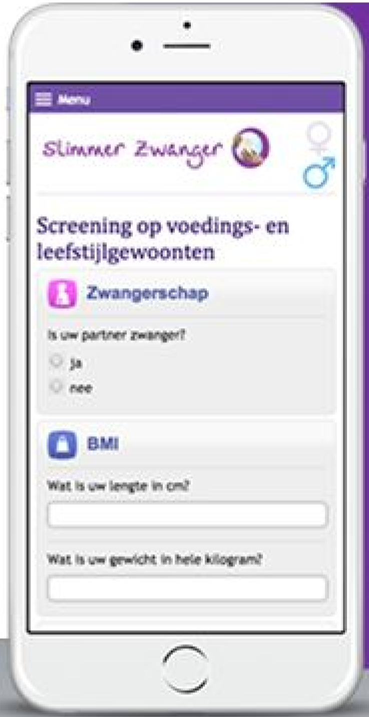

mHealth program:

Improves nutrition and lifestyle in women and men contemplating pregnancy (or being pregnant) through personal coaching using e-mail and text messaging during 26 weeks.

www.slimmerzwanger.nl
www.smarterpregnancy.co.uk

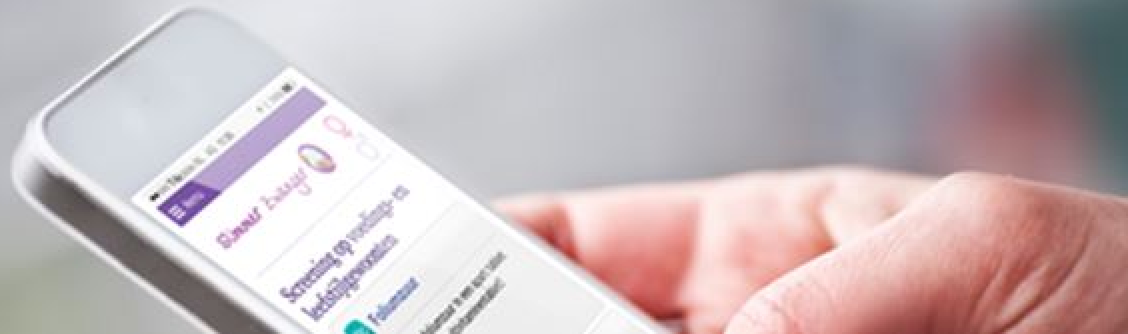

Supplement: Multimedia Appendix 1 [file mhealth_v7i4e11664_app1.pdf]
