# Supplementary figures and images for: Neighborhood Deprivation and the Effectiveness of Mobile Health Coaching to Improve Periconceptional Nutrition and Lifestyle in Women: Survey in a Large Urban Municipality in the Netherlands
Source: JMIR Mhealth Uhealth. 2019 Apr 11;7(4):e11664. doi: 10.2196/11664 (PMC6482404; doi:10.2196/11664)

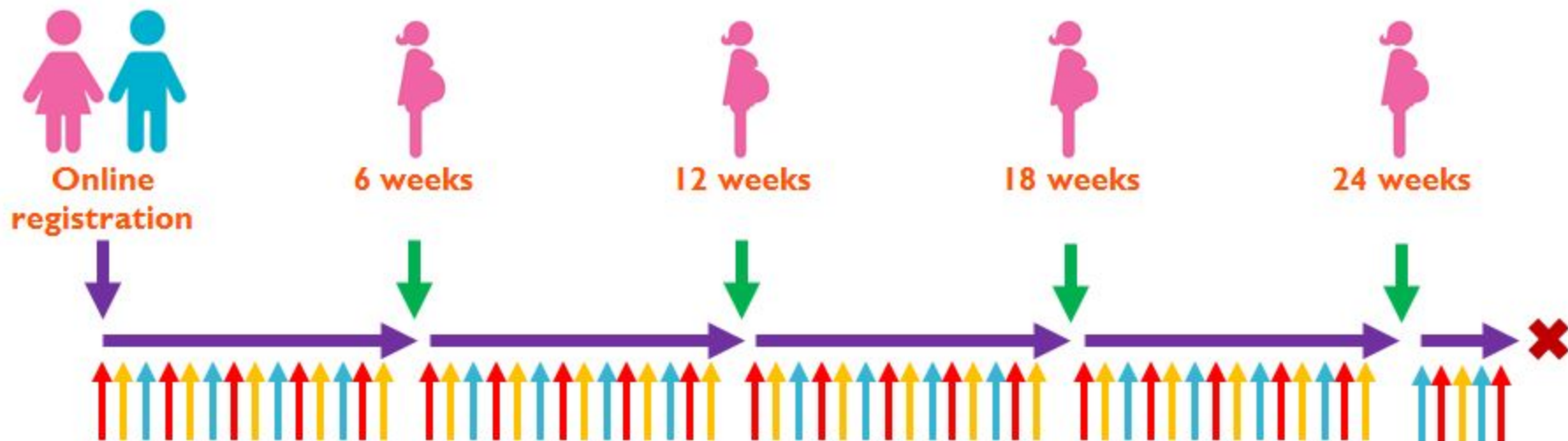

## Legend

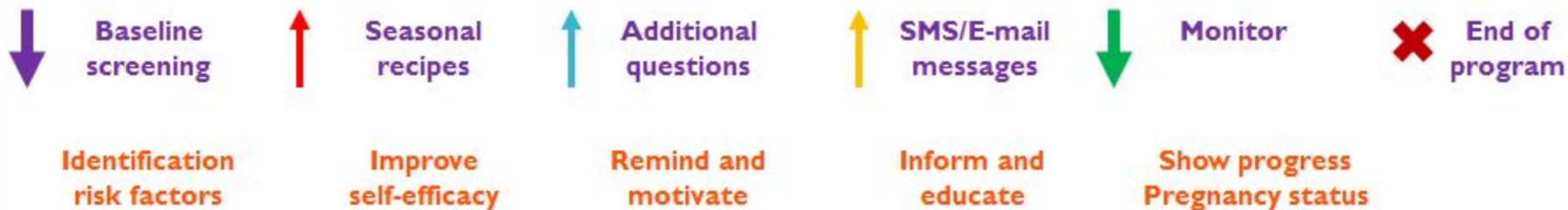

Supplement: Multimedia Appendix 2 [file mhealth_v7i4e11664_app2.pdf]
